# Rapid tranquillisation for psychiatric in-patients with a diagnosis of personality disorder: under-recognised issue

**DOI:** 10.1192/bjo.2025.10052

**Published:** 2025-08-01

**Authors:** Carol Paton, Mike J. Crawford, Matthew Hartley, Clive E. Adams, Elena M. Edokpolor Pernia, Olivia Rendora, Thomas R. E. Barnes

**Affiliations:** Prescribing Observatory for Mental Health, Royal College of Psychiatrists, London, UK; Division of Psychiatry, Imperial College London, London, UK; Behavioural & Developmental Psychiatry Clinical Academic Group, South London and Maudsley NHS Foundation Trust, London, UK; Mental Health Services Research. University of Nottingham, Nottingham, UK

**Keywords:** Personality disorder, acutely disturbed behaviour, rapid tranquillisation, self-harm

## Abstract

**Background:**

Clinical guidelines for personality disorder emphasise the importance of patients being supported to develop psychological skills to help them manage their symptoms and behaviours. But where these mechanisms fail, and hospital admission occurs, little is known about how episodes of acutely disturbed behaviour are managed.

**Aims:**

To explore the clinical characteristics and management of episodes of acutely disturbed behaviour requiring medication in in-patients with a diagnosis of personality disorder.

**Method:**

Analysis of clinical audit data collected in 2024 by the Prescribing Observatory for Mental Health, as part of a quality improvement programme addressing the pharmacological management of acutely disturbed behaviour. Data were collected from clinical records using a bespoke proforma.

**Results:**

Sixty-two mental health Trusts submitted data on 951 episodes of acutely disturbed behaviour involving patients with a personality disorder, with this being the sole psychiatric diagnosis in 471 (50%). Of the total, 782 (82%) episodes occurred in female patients. Compared with males, episodes in females were three times more likely to involve self-harming behaviour or be considered to pose such a risk (22% and 70% respectively: *p* < 0.001). Parenteral medication (rapid tranquillisation) was administered twice as often in episodes involving females than in males (64 and 34% respectively: *p* < 0.001).

**Conclusions:**

Our findings suggest that there are a large number of episodes of acutely disturbed behaviour on psychiatric wards in women with a diagnosis of personality disorder. These episodes are characterised by self-harm and regularly prompt the administration of rapid tranquillisation. This has potential implications for service design, staff training, and research.

There are two National Institute for Health and Care Excellence (NICE) guidelines that address the clinical management of patients with a personality disorder. The first focuses on borderline personality disorder^1^ and recommends avoiding admission to psychiatric wards where possible, although if this proves to be necessary, the advice is to move from compulsory treatment to management on a voluntary basis at the earliest opportunity. Short-term treatment with an oral sedative medication such as an antihistamine is recommended during periods of crisis. The second NICE guideline^2^ focuses on antisocial personality disorder, noting that there are few evidence-based interventions and that pharmacological interventions should be restricted to the treatment of comorbid conditions. Neither guideline directly addresses the pharmacological management of acutely disturbed behaviour, which may manifest as deliberate self-harm, in in-patient settings, although there are two further NICE clinical guidelines that are relevant to this. The NICE guideline for the management of self-harm^3^ makes a number of recommendations relating to the risk assessment of those who may self-harm, and the aftercare of those individuals who have self-harmed although it does not explicitly consider management strategies during episodes of actual or threatened self-harm, while the NICE guideline for the management of violence and aggression^4^ makes evidence-based recommendations relating to the use of restrictive interventions where a person is aggressive but again does not directly address self-harm. Patients with a personality disorder whose problems are severe and refractory are relatively commonly admitted to psychiatric beds during crises: hospital admission statistics suggest that personality disorder is the primary reason for admission to mental health services in just under one in twenty cases in England and Wales.^5^ Diagnoses that are commonly comorbid with personality disorder such as mental disorders due to psychoactive substance use, mood disorders, and anxiety, were the primary reasons for admission in around a quarter, fifth and tenth of cases, respectively.^5^

The Prescribing Observatory for Mental Health (POMH) has been conducting a quality improvement programme that focuses on the pharmacological management of acutely disturbed behaviour in psychiatric in-patient settings; this has been described in detail elsewhere.^6,7^ Given that clinical guidelines do not directly address the pharmacological management of crises in patients with a diagnosis of personality disorder beyond short-term treatment with an oral sedative medication,^1,8^ and that patients with personality disorder are often admitted to psychiatric wards, we took the opportunity to analyse audit data collected in the context of this programme to explore how medication is used to manage acutely disturbed behaviour in such patients.

## Method

The Prescribing Observatory for Mental Health (POMH) is based at the Centre for Quality Improvement at the Royal College of Psychiatrists in the UK. For the past 20 years, POMH has been conducting audit-based, quality improvement programmes addressing prescribing practice in UK mental health services. Briefly, for each quality improvement programme, audit standards are derived from evidence-based clinical guidelines and endorsed by an expert working group. For each programme a bespoke audit tool is developed and refined in discussion with member Trusts. An audit period is set (a 2-month window) during which data are entered online. After each audit, every participating Trust receives a customised report that includes data on their clinical performance, benchmarked against evidence-based practice standards.^9,10^ The majority of UK mental health Trusts/healthcare organisations are POMH members and participate in these programmes.

In 2024, all POMH member Trusts (*n* = 64) were invited to participate in a clinical audit in the context of a quality improvement programme addressing the pharmacological management of episodes of acutely disturbed behaviour in adult psychiatric in-patients. Eligible for inclusion was practice related to any in-patient on an acute adult or forensic psychiatric ward who had received either oral or parenteral medication to manage such an episode during the period from 1st March to the 30th April 2024. Neither ethical approval^11^ nor patient consent is required for such audit-based, quality improvement initiatives.

A standardised, bespoke audit tool was used, which had been designed to gather relevant information, principally relating to performance against several evidence-based practice standards, from clinical records.^6,7^ The audit standards for this quality improvement programme, which are not reported on here, related to the quality of care planning and physical health monitoring after episodes of rapid tranquillisation.

The audit data collected for each episode that are relevant to the aims of this paper included the following: patient age, sex, ethnicity, psychiatric diagnosis and legal status; type of ward providing care; regularly prescribed psychotropic medication; non-pharmacological interventions used prior to administering additional medication to manage the episode of acutely disturbed behaviour; and the medication administered to manage the episode. Further, the person completing the data collection tool was asked to review the documented description of the behavioural disturbance at the time of the episode and, if this were detailed enough to allow it, assess the level of disturbance according to the Behavioural Activity Rating Scale (BARS) descriptors (1 = Difficult or unable to arouse, 2 = Asleep but responds normally to physical contact, 3 = Drowsy but appears sedated, 4 = Quiet and awake, normal level of activity, 5 = Signs of overt physical or verbal activity, calms down with instructions, 6 = Extremely or continuously active not requiring restraint, 7 = Violent, requires restraint).^12^ In this paper, we are only reporting on the subsample of episodes that involved patients with a diagnosis of personality disorder.

To avoid the potential for data to be submitted for multiple administrations of medication in the same patient over a short period of time, Trusts were asked to limit submissions to one completed audit tool for each episode of acutely disturbed behaviour unless these episodes were separated by at least 7 days. Further, submitted data were examined and any cases that were potentially duplicates (i.e. matched on all variables) were discussed with the submitting Trust and removed from the dataset as necessary.

### Data analysis

Data were submitted on-line using Formic clinical audit software, version 5.7.1 (in Windows 11, Formic Healthcare; see (https://www.formic.com/), and analysed using SPSS version 26.0.0.0 (in Windows 11, IBM; see https://www.ibm.com/support/pages/downloading-ibm-spss-statistics-26). Simple descriptive statistics were used to describe the demographic and clinical characteristics of the audit sample. *χ* ^2^ texts were used to explore differences between sub-samples.

As the data submitted were pseudonymous, it was not possible to identify the exact number of patients for whom data were submitted; for example episodes separated by more than a week where regular medication had been changed would not be identified as duplicates during the data cleaning process. The data are therefore reported at the level of episodes of disturbed behaviour rather than individual patients.

## Results

Data were submitted by 62 trusts for 3640 in-patient episodes of acutely disturbed behaviour managed by the administration of medication. Of these epsiodes, 951 (26%) involved patients with a diagnosis of a personality disorder and the demographic and clinical characteristics of these patients are shown in Table 1. In 480 (50%) of these 951 episodes, the patient involved had a co-morbid psychiatric diagnosis: the most common co-morbid conditions were a schizophrenia spectrum disorder in 156 cases (33%), an affective disorder in 143 (30%), a neurotic disorder in 135 (28%), a disorder of psychological development in 97 (20%) and psychoactive substance misuse in 74 (15%).

**Table 1:** Demographic and clinical characteristics of the female and male in-patients with a personality disorder diagnosis involved in episodes of acutely disturbed behaviour treated with medication

	Male patient episodes *n* = 169 *n* (%)	Female patient episodes *n* = 782 *n* (%)
Personality disorder diagnosis		
As the sole psychiatric diagnosis	58 (34)	413 (53)
With ≥1 co-morbid psychiatric diagnosis	111 (66)	369 (47)
Ethnicity		
White/White British	140 (83)	644 (82)
Black/Black British	13 (8)	16 (2)
Asian/Asian British	5 (3)	11 (1)
Mixed or other	6 (4)	19 (2)
Not available	5 (3)	92 (12)
Age in years		
Median (range)	33 (19–78)	27 (16–89)
18–25	42 (25)	345 (44)
26–35	59 (35)	281 (36)
36–45	32 (19)	83 (11)
46–55	19 (11)	45 (6)
56–65	14 (8)	18 (2)
Over 65	3 (2)	7 (1)
Psychiatric diagnoses co-morbid with personality disorder (ICD-10 categories)		
Organic disorder (F00–09)	1 (1)	2 (0)
Disorder due to psychoactive substance use (F10–19)	32 (19)	42 (5)
Schizophrenia spectrum disorder (F20–29)	61 (36)	95 (12)
Affective disorder (F30–39)	17 (10)	126 (16)
Neurotic, stress-related and somatoform disorders (F40–49)	3 (2)	132 (17)
Behavioural syndromes associated with physiological disturbances and physical factors (F50–59)	1 (1)	23 (3)
Learning disability (F70–79)	15 (9)	39 (5)
Disorders of psychological development (F80–89)	15 (9)	82 (10)
Behavioural and emotional disorders with onset usually occurring in childhood and adolescence (F90–98)	12 (7)	42 (5)
Unspecified disorder (F99)	1 (1)	4 (1)
MHA status		
Informal	30 (18)	70 (9)
Section 2	34 (20)	165 (21)
Section 3	66 (39)	487 (62)
Forensic section	11 (7)	8 (1)
Other section	28 (17)	52 (7)
Clinical service		
Acute adult psychiatric ward	95 (56)	578 (74)
Psychiatric Intensive care unit	24 (14)	93 (12)
Low secure/locked psychiatric ward	24 (14)	48 (6)
Forensic psychiatric ward	26 (15)	63 (8)

Of the 951 episodes in patients with a diagnosis of personality disorder, 782 (82%) involved females. Females were more likely than males to have personality disorder as their sole psychiatric diagnosis (53 *v*. 34%, *χ* ^*2*^ = 19.014, d.f. = 1, *p* < 0.001), be 25 years of age or younger (45 *v*. 25%, *χ* ^2^ = 22.179, d.f. = 1, *p* < 0.001), have a comorbid diagnosis of an anxiety spectrum disorder (17 *v*. 2%, *χ* ^*2*^ = 26.030, d.f. = 1, *p* < 0.001), be detained under Section 3 of the Mental Health Act (62 *v*. 39% *χ* ^*2*^ = 30.796, d.f. = 1, *p* < 0.001) and be cared for on an acute adult ward (74 *v*. 56%, *χ* ^*2*^ = 21.046, d.f. = 1, *p* < 0.001). Males were more likely to have a comorbid substance misuse (19 *v*. 5%, *χ* ^*2*^ = 35.631, d.f. = 1, *p* < 0.001) or schizophrenia spectrum disorder diagnosis (36 v 12%, *χ* ^*2*^ = 58.111, d.f. = 1, *p* < 0.001). Further details relating to the demographic and clinical characteristics of the female and male subsamples are shown in Table 1.

The pattern of prescribing with respect to regular psychotropic medications was similar for females and males with the exception that the former were more likely to be prescribed antidepressant medication (*n* = 451, 58% *v*. *n* = 61, 36%; *χ* ^2^ = 26.035, d.f. = 1, *p* < 0.001). As shown in Table 2, the pattern of prescribing of regular psychotropic medication was very similar in those with personality disorder as a sole psychiatric diagnosis and those who had a comorbid psychiatric illness.

**Table 2:** Regularly prescribed psychotropic medication for the patients with a sole diagnosis of personality disorder or with a comorbid mental illness involved in episodes of acutely disturbed behaviour treated with medication

	Episodes of acutely disturbed behaviour	
Psychotropic medication regularly prescribed	Patients with personality disorder alone *n* = 471 *n* (%)	Patients with comorbid psychiatric illness *n* = 480 *n* (%)
Oral antipsychotic		
**Any oral antipsychotic**	**299 (63)**	**309 (64)**
Most commonly prescribed		
Quetiapine	95 (20)	102 (21)
Aripiprazole	83 (18)	63 (13)
Olanzapine	31 (7)	79 (16)
Long-acting injectable (LAI) antipsychotic		
**Any LAI**	**111 (24)**	**134 (28)**
Most commonly prescribed		
Zuclopenthixol	65 (14)	66 (14)
Haloperidol	10 (2)	24 (5)
Flupentixol	15 (3)	11 (2)
**Any antipsychotic medication**	**380 (81)**	**400 (83)**
Benzodiazepine		
**Any benzodiazepine**	**158 (34)**	**198 (41)**
Most commonly prescribed		
Clonazepam	96 (20)	91 (19)
Diazepam	44 (9)	65 (14)
Lorazepam	18 (4)	40 (8)
Other psychotropic medication		
Most commonly prescribed		
Antidepressant	260 (55)	252 (53)
Mood stabiliser	109 (23)	127 (26)
Promethazine	40 (8)	58 (12)
Anticholinergic	45 (10)	44 (9)
None of the above	33 (7)	19 (4)

### The clinical presentation of the episodes of acutely disturbed behaviour in the in-patients with a diagnosis of personality disorder

For 784 (82%) episodes, there was sufficient information in the clinical records relating to the level of behavioural disturbance at the time additional medication was administered to allow a BARS^11^ descriptor to be allocated. In 448 (57%) of these episodes, the patient was considered to be violent, requiring restraint (BARS score of 7) and in a further 186 (24%) was assessed as being extremely or continuously active but not requiring restraint (BARS score of 6). There was no difference in the profile of BARS scores in the episodes involving males and females.

**Fig. 1:**
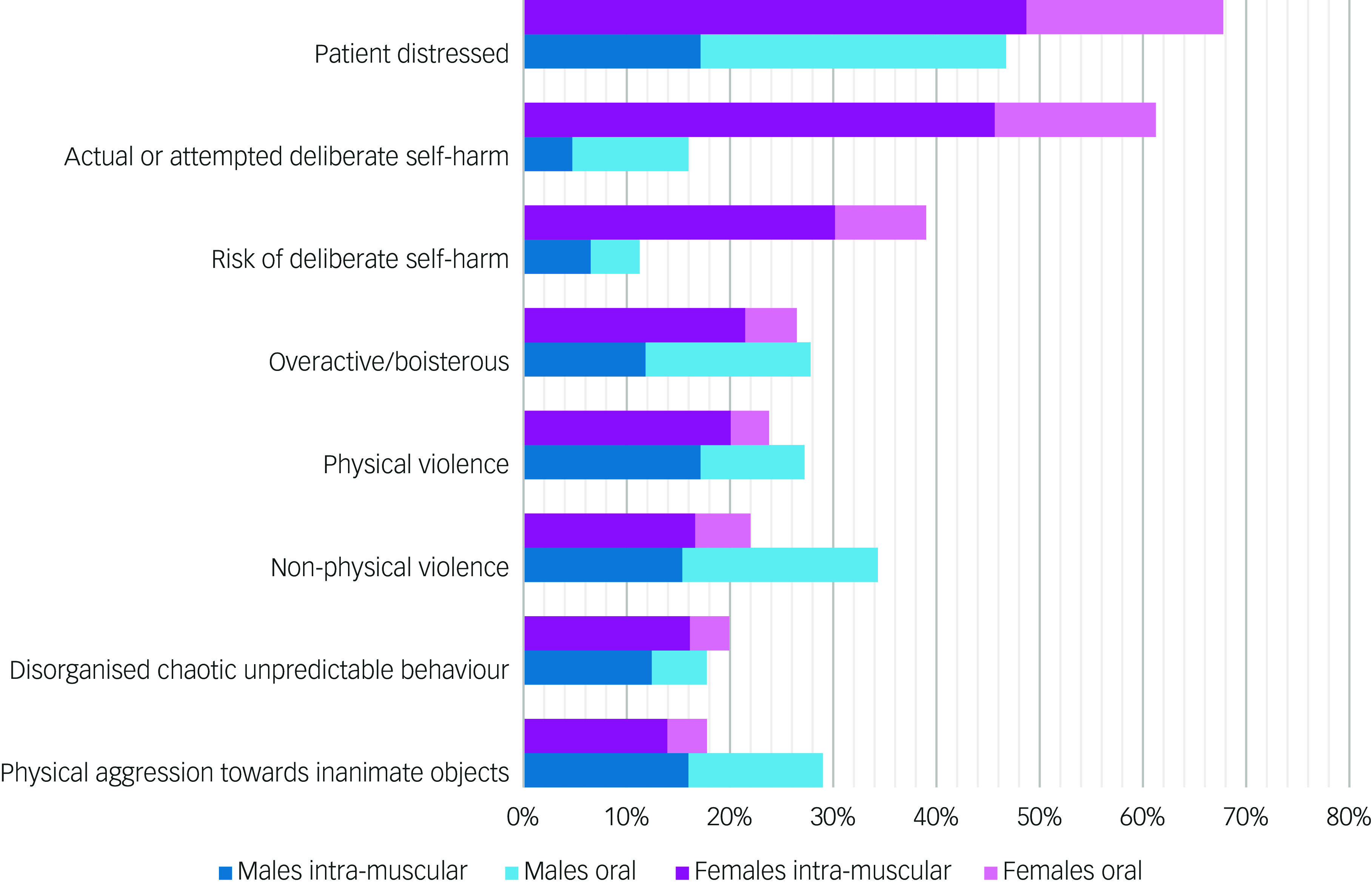
Nature of acutely disturbed behaviour documented at the time parenteral or oral medication was administered: episodes in female (*n* = 782) and male (*n* = 169) patients.

As can be seen in Fig. 1, the most common target for psychotropic medication was patient distress followed by self-harm and violence. The symptoms/behaviours reported in fewer than 10% of episodes are not shown in this figure; these include hallucinations, self-isolation, expression of delusional ideas, thought disorder and sexual disinhibition.

Compared with males, episodes involving females were more likely to include self-harming behaviour or an immediate risk of self-harm (*n* = 38, 22% males *v*. *n* = 546, 70% females; χ^2^ = 131.393, d.f. = 1, *p* < 0.001). However, episodes involving males were more likely than those involving females to present with physical or non-physical violence or violence against inanimate objects (*n* = 90, 53% males *v*. *n* = 315, 40% females; χ^2^ = 9.566, d.f. = 1, *p* = 0.002).

### The clinical management of the episodes of acutely disturbed behaviour in the in-patients with a diagnosis of personality disorder

**Fig. 2:**
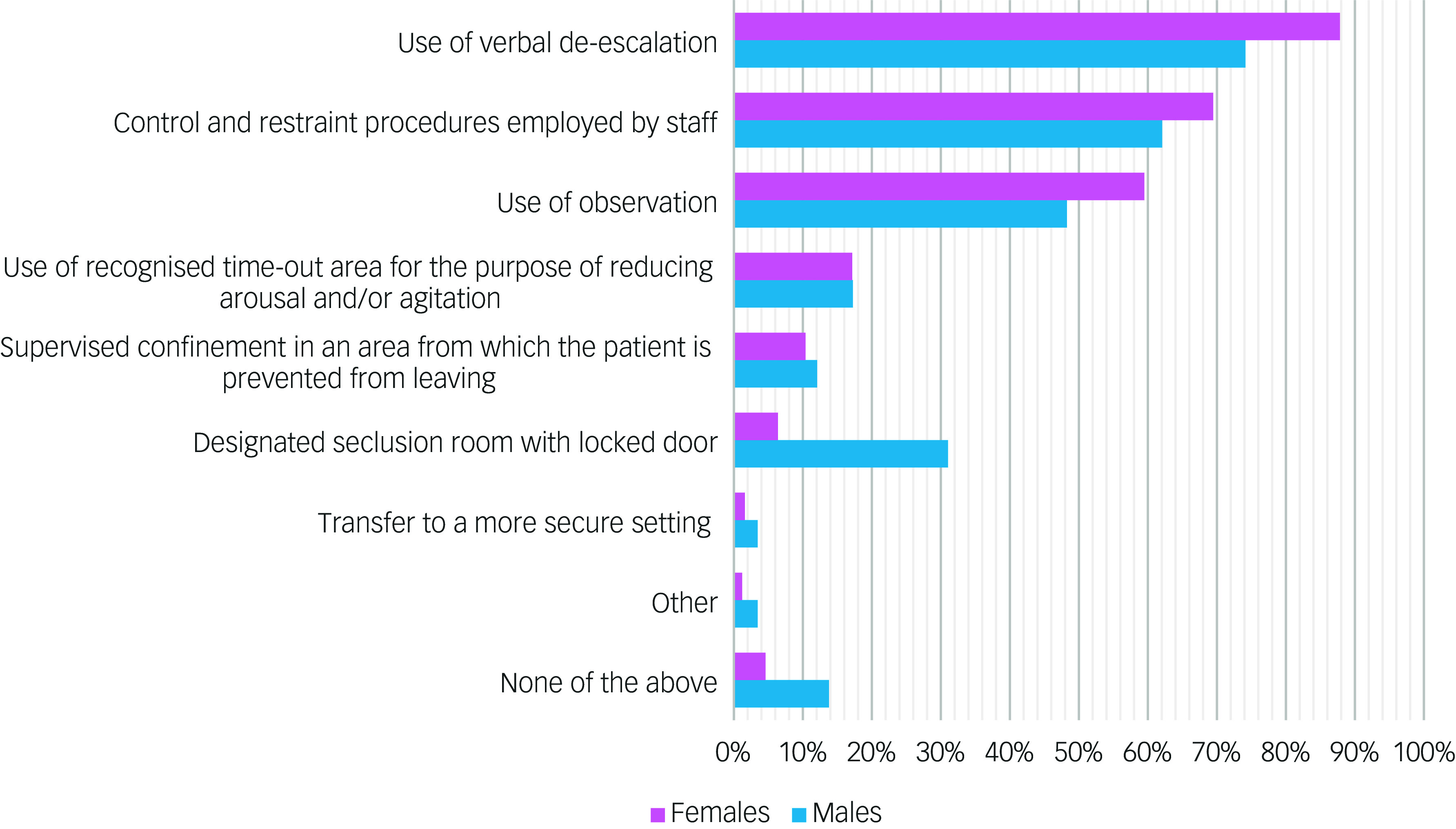
Documented non-pharmacological interventions prior to administering parenteral medication for acutely disturbed behaviour: episodes in female (*n* = 501) and male (*n* = 58) patients.

In the 559 episodes where parenteral medication was used (rapid tranquillisation), this was after verbal de-escalation had been tried in the vast majority of cases. As can be seen in Fig. 2, control and restraint techniques and the use of observation had been used in more than half of cases.

The episodes of acutely disturbed behaviour were managed with oral medication in 392 (41%) cases and parenteral medication in the remaining 559 (59%). Rapid tranquillisation was administered in 501 (64%) episodes involving female patients and 58 (34%) involving males (*χ* ^2^ = 50.753, d.f. = 1, *p* < 0.001). Fig. 1 shows the profile of use of parenteral and oral medication to manage the episodes of acutely disturbed behaviour in the female and male patients. Details of the parenteral medication administered for rapid tranquillisation are shown in Table 3.

**Table 3:** Intra-muscular medication administered for episodes of acutely disturbed behaviour in 501 female and 58 male in-patients with a diagnosis of personality disorder

Intra-muscular medication administered for the episode	Females *n* = 501^a^ *n* (%)	Males *n* = 58 *n* (%)
Benzodiazepine alone	292 (58)	30 (52)
Promethazine alone	101 (20)	3 (5)
Antipsychotic alone	34 (7)	10 (17)
Benzodiazepine and promethazine	36 (7)	4 (7)
Benzodiazepine and antipsychotic	18 (4)	6 (10)
Promethazine and antipsychotic	15 (3)	5 (9)
Benzodiazepine, promethazine, and antipsychotic	2 (<1%)	–

a. Data were missing in three cases.

## Discussion

Our clinical audit, conducted nationally, collected information on the pharmacological treatment of episodes of acutely disturbed behaviour occurring in acute adult and forensic in-patient settings. In just over a quarter of episodes, the patient had a diagnosis of personality disorder and this was the sole psychiatric diagnosis in half of these cases. The vast majority of such episodes involved females and the most common clinical features associated with the administration of the additional psychotropic medication were deliberate self-harm and patient distress. Episodes involving females were more likely to result in the administration of parenteral medication than episodes involving males. Thus, our findings suggest that there are a relatively large number of episodes of acutely-disturbed behaviour occurring on UK adult psychiatric wards that involve females with a diagnosis of personality disorder, and these regularly prompt the administration of parenteral medication.

### Clinical and demographic characteristics of the patients with a diagnosis of personality disorder

In the 951 episodes in in-patients with a diagnosis of personality disorder exhibiting acutely disturbed behaviour, the vast majority involved patients who were detained in hospital under mental health legislation and were relatively young, with a median age of 30 years; these findings are consistent with those of previous national audits of the use of RT that have included all episodes, irrespective of the patients’ psychiatric diagnoses.^6,7^

Just over four-fifths of the reported episodes occurred in females, a finding that is consistent with the routinely collected data submitted by mental health services to NHS England,^13^ where the most recent data from August 2024 show that females in general were four times more likely to receive rapid tranquillisation than males: sixteen administrations per 1000 occupied bed days for females and four for males. Our data suggest that the females who received rapid tranquillisation were not more behaviourally disturbed than males, but rather it was the nature of the clinical presentation that had prompted the use of additional pharmacological interventions.

People with personality disorder are known to have high levels of psychiatric co-morbidity; for example, the prevalence of mood and anxiety disorders are high in patients with borderline personality disorder, while depression and substance misuse disorders are commonly seen in those with paranoid personality disorder.^14^ Consistent with this, around half the episodes in our sample involved patients who had a diagnosed comorbid psychiatric illness: these were most commonly mood and anxiety spectrum disorders in the female patients and schizophrenia and substance misuse in the males. However, in half of the episodes there was no comorbid psychiatric diagnosis, suggesting that the acutely disturbed behaviour was associated with a diagnosis of personality disorder.

The National Institute for Health and Care Excellence (NICE) recommends that people with borderline or antisocial personality disorders are only prescribed antipsychotic or sedative medication for short-term crisis management or treatment of comorbid conditions.^1,8^ However, the prescription of regular background psychotropic medication was common in episodes involving patients in our sample who had a sole psychiatric diagnosis of personality disorder, with antipsychotic, benzodiazepine, antidepressant and mood stabiliser medications being prescribed in around four-fifths, two-fifths, half and a quarter of episodes respectively. The pattern of prescribing was very similar in those episodes involving patients with a diagnosed co-morbid psychiatric illness. These findings are consistent with those in a large national audit of prescribing for emotionally unstable personality disorder conducted by POMH^15^ and suggest that, rather than following the recommendations in clinical guidelines,^1,8^ clinicians continue to take a symptom-targeted approach to prescribing for people with personality disorder.

### Clinical presentation of the episodes of acutely disturbed behaviour in patients with a diagnosis of personality disorder

Where the level of behavioural disturbance, at the point that additional psychotropic medication was administered, was documented sufficiently well to allow a BARS^11^ descriptor to be allocated, the patient was at least ‘extremely or continuously active’ (BARS rating of 6 or 7) in more than four-fifths of cases. However, while the overall level of behavioural disturbance was very similar in the female and male patients, the most frequently documented symptoms and behaviours differed between the sexes. Our data suggest that, compared with the male patients, episodes involving females were much more likely to involve distress that was directed internally in the form of self-harm. In contrast, episodes of disturbed behaviour in male patients were much more likely to involve externally directed aggression, towards others or property. These different profiles in females and males replicate the findings in a previous national audit that addressed in-patient episodes where additional psychotropic medication was used to manage acutely disturbed behaviour, irrespective of the psychiatric diagnosis;^6^ these findings have implications for staff training and local guidelines for the management of acutely disturbed behaviour. There are also potential implications for care pathways: that women with a personality disorder manifest their distress through self-harm, and that the risks this poses are severe enough to result in in-patient psychiatric care and the relatively common use of parenteral medication, raises the question of whether additional training and resources are needed in community teams to prevent and manage crises.

### Clinical management of the episodes of acutely disturbed behaviour in patients with a diagnosis of personality disorder

Parenteral medication was prescribed for two-thirds of episodes involving female patients compared with one-third of those involving males. The medication administered included a benzodiazepine in over two-thirds of cases, with no difference between the sexes. However, intra-muscular promethazine, a sedative antihistamine, was one and a half times more likely to be administered to manage episodes involving females patients than males, perhaps influenced by the recommendation by NICE^1^ that such a medication, albeit administered orally, may be appropriate for use short term during a crisis. In contrast, episodes involving male patients were almost three times more likely than those in females to be managed with intra-muscular antipsychotic medication, with the most likely reason for this difference being the higher prevalence of comorbid psychotic illness in the former. The medication administered to manage the episode of acutely-disturbed behaviour was almost always in addition to regularly prescribed psychotropic medication, potentially resulting in complex polypharmacy and the associated side-effect burden.

De-escalation strategies, control and restraint techniques and the use of observation were used in managing episodes of acutely-disturbed behaviour in the majority of cases, with only modest differences between the sexes. Compared with the females, the management of episodes involving males was four times more likely to involve the use of seclusion. The relatively high levels of externally directed aggression exhibited by males, along with the likelihood that seclusion facilities are more likely to be available in clinical settings that care for males, may explain this finding.

#### Implications for clinical service delivery

In England, services for people with personality disorder have expanded over the last 20 years.^16^ In addition to delivering evidence-based psychological treatments, dedicated personality disorder services usually provide consultation and training services to support other teams working with people with personality disorder.^17^ To date, most of this work has focused on supporting staff in community-based services, but the results of this audit highlight the need to provide support for in-patient staff. For example, a report of a service reorganisation suggested that levels of restraint and seclusion can be reduced in in-patient services that deliver psychological skills to people with personality disorder.^18^ Such pioneering work could give foundation to the fair testing of different modes of delivery within the context of pragmatic randomised trials.

### Self-harming behaviour during episodes of acutely-disturbed behaviour in patients with a diagnosis of personality disorder

The major difference in clinical presentation between the sexes was not the overall level of behavioural disturbance but the nature of that disturbance. The finding that a much higher proportion of episodes in the female patients with a diagnosis of personality disorder involved deliberate self-harm, suggests that such behaviour is a key prompt for the use of rapid tranquillisation.

#### Implications for clinical service delivery

Strategies to prevent and manage self-harming behaviour are based on behavioural chain analysis, which is a component of dialectical behaviour therapy (DBT); the aim is to understand the antecedents, the behaviour itself and the consequences of this behaviour and support the individual to process the antecedents and find a way of managing these that does not involve engaging in life-threatening behaviours: essentially, to develop internal control mechanisms.^19^ However, while patients may be able to engage in such an approach while their mental state is reasonably stable, in a period of crisis the ability to do this may be more limited. At such times, distress tolerance techniques, including distraction, self-soothing and behavioural interventions may enable people to resist the urge to self-harm.^20,21^ Nursing staff may perceive that it is too risky to rely wholly on defusing the situation, particularly if the individual has in their possession an object that can cause serious harm, such as a broken cup or a ligature, and staff may feel compelled to move on quickly to the use of control and restraint and parenteral medication in order to keep the person safe. However, such a decision may have its own drawbacks, for example, RT involves removing control from the patient and transferring this to staff, a message that may be potentially confusing for patients. Further, women who self-harm often have a history of abuse^20^ and may perceive being restrained and having their clothing moved to administer parenteral medication into the buttock or thigh as provocative and re-traumatising. While it is clearly challenging to keep a person safe while causing minimal harm, the NICE guideline that focuses on service user experience in adult mental health services^22^ offers a way forward by emphasising the importance of co-design, with patients having a voice in the way service models are tested and delivered. The management of self-harm in in-patient settings would be a worthy focus for such co-design initiatives.

### Limitations of the study

The audit data collected were derived solely from the clinical records maintained by staff working on in-patient wards. Further, information on sub-types of personality disorder was not collected; however, given that all the episodes of disturbed behaviour occurred in acute adult in-patient settings, it is likely that the personality disorders being treated were severe, complex and refractory to standard treatments. Finally, the audit data were submitted by a large number of NHS Trusts, predominantly from acute adult and forensic mental health in-patient services, so while the findings are likely to be generalisable across these settings in the UK, they may not be generalisable to other countries or other healthcare systems.

### Clinical implications

Our findings suggest that there are a relatively large number of episodes of acutely disturbed behaviour on adult psychiatric wards that involve females with a diagnosis of personality disorder. These episodes are often characterised by self-harm and frequently managed with rapid tranquillisation. Given the potential implications for service design, staff training, and research, this is a clinical issue that warrants more attention in the literature, as well as coverage in guidelines and training programmes addressing the management of acutely disturbed behaviour.

## Data Availability

The aggregated data-set that supports these findings is not openly available. Membership agreements between Prescribing Observatory for Mental Health (Prescribing Observatory for Mental Health Prescribing Observatory for Mental Health (POMH) and participating mental health services state that each mental health service owns its own data-set and that this will not be shared by POMH with any third party. POMH is restricted to reporting on analyses based on the aggregated national data-set.
